# Does delivering chest compressions to patients who are not in cardiac arrest cause unintentional injury? A systematic review

**DOI:** 10.1016/j.resplu.2024.100828

**Published:** 2024-11-23

**Authors:** Frances Williamson, Pek Jen Heng, Masashi Okubo, Abel Martinez Mejias, Wei-Tien Chang, Matthew Douma, Jestin Carlson, James Raitt, Therese Djärv

**Affiliations:** a Emergency and Trauma Centre|Trauma Service, Royal Brisbane and Women’s Hospital, Australia; b Department of Emergency Medicine, Sengkang General Hospital, Singapore, Singapore; c Department of Emergency Medicine, University of Pittsburgh School of Medicine, United States; d Department of Pediatrics, Terrassa University Hospital, Consorci Sanitari of Terrassa, CST, Barcelona, Spain; e National Taiwan University, Taipei, Taiwan; f Department of Critical Care Medicine, University of Alberta, Canada; g Allegheny Health Network Institutional Review Board, United States; h Thames Valley Air Ambulance, Stokenchurch, UK; i Department of Medicine Solna, Karolinska Institute and Division of Acute and Reparative Medicine, Karolinska University Hospital, Sweden

**Keywords:** Cardiac arrest, Out-of-hospital cardiac arrest, Cardiopulmonary resuscitation, Bystander, harm, adverse events

## Abstract

**Background:**

Chest compressions are life-saving in cardiac arrest but concern by layperson of causing unintentional injury to patients who are not in cardiac arrest may limit provision and therefore delay initiation when required.

**Aim:**

To perform a systematic review of the evidence to identify if; among patients not in cardiac arrest outside of a hospital, does provision of chest compressions from a layperson, compared to no use of chest compressions, worsen outcomes.

**Method:**

We searched Medline (Ovid), Web of Science Core Collection (clarivate) and Cinahl (Ebsco). Outcomes included survival with favourable neurological/functional outcome at discharge or 30 days; unintentional injury (e.g. rib fracture, bleeding); risk of injury (e.g. aspiration). ROBINS-I was used to assess for risk of bias. Grading of Recommendations, Assessment, Development and Evaluation methodology was used to determine the certainty of evidence. (PROSPERO registration number: CRD42023476764).

**Results:**

From 7832 screened references, five observational studies were included, totaling 1031 patients. No deaths directly attributable to chest compressions were reported, but 61 (6 %) died before discharge due to underlying conditions. In total, 9 (<1%) experienced injuries, including rib fractures and different internal bleedings, and 24 (2 %) reported symptoms such as chest pain. Evidence was of very low certainty due to risk of bias and imprecision.

**Conclusion:**

Patients initially receiving chest compressions by a layperson and who later were determined by health care professionals to not be in cardiac arrest rarely had injuries from chest compressions.

## Introduction

Delivery of high-quality chest compressions is a key step in the chain of survival for patients in cardiac arrest and immediate cardio-pulmonary resuscitation (CPR) initiated by laypersons is associated with improved outcomes.^1^ However, there are documented adverse effects of CPR in patients being in cardiac arrest in which injuries are identified in 30–45 % of cases including most frequently rib and sternal fractures and lung and abdominal organ injuries.^2^ There may be a reluctance amongst laypersons to initiate CPR for fear of causing harm^3^, ^4^ and a better understanding of the potential injuries could help encourage laypersons to initiate CPR, either as chest compression only or as chest-compressions with ventilation.

In 2020, the International Liaison Committee on Resuscitation (ILCOR) published a consensus on science with treatment recommendations (CoSTR), and noted very-low-certainty evidence, concluding that laypersons should initiate CPR for presumed cardiac arrest as the low risk of harm to patients not in cardiac arrest was outweighed by the potential patients in cardiac arrest.^5^

Knowing the possible injuries of chest compressions in patients who are not in cardiac arrest is of great importance in making recommendations for laypersons. This systematic review was performed on behalf of the ILCOR First Aid Task Forces and was designed to answer the question: Among patients not in cardiac arrest outside of a hospital (P), does provision of chest compressions from laypersons (I), compared to no use of chest compressions (C), change the outcomes of survival with a favorable neurological/functional outcome at discharge, 30 days, 60 days, 180 days, and/or 1 year; any unintentional injury (e.g. rib fracture, major bleeding) or risk of injury (e.g. aspiration)?

### Methods

ILCOR uses a continuous evidence process to evaluate evidence and develop treatment recommendations for resuscitation and relevant first aid topics culminating in the publication of a CoSTR. This systematic review was conducted in accordance with ILCOR standards based on the Cochrane Handbook for Systematic Reviews of Interventions,^6^, and reporting occurred through the Preferred Reporting Items for Systematic Reviews and Meta-Analyses (PRISMA) checklist.^7^, ^8^The protocol for this systematic review was registered with PROSPERO (registration number: CRD42023476764). Changes from the protocol was not searching the Cochrane database after dialog with information specialists and naming of outcomes, the registered “harm” of CPR was changed to “unintentional physical injury” since harm might be associated to something intentional.

No ethical approval was sought for this systematic review.

### Selection criteria

The following inclusion and exclusion criteria were used to select published articles:

Within this systematic review, the term “not in cardiac arrest” refers to a person or patient who was judged by Emergency Medical Services (EMS) at their arrival not to be in a cardiac-arrest (i.e. EMS record documented the presence of a palpable pulse, if the record documented a working assessment other than cardiac arrest, EMS did not perform CPR etc.). Precise definitions of each article are included in Supplementary table A. We defined “layperson” as a person performing CPR not being part of an organized medical team. “Chest compressions” were defined as chest compressions done by a layperson, regardless of if it was via instructions from a dispatcher centre or not.

Population: Studies in adults and children who are not in cardiac arrest (CA) outside of a hospital (OHCA). To be “not in cardiac arrest” was defined as in included studies, most often via judgement by EMS at their arrival. No exclusion criteria were used.

Intervention: Studies where the provision of chest compressions, regardless if they were combined with rescue breaths or not, was done by lay providers. Further, studies were included regardless if laypersons were trained to do CPR and whether it was via instructions from a dispatcher centre or not. Excluded: Studies where laypersons only provided ventilations and no chest compressions.

Comparison: Studies where no chest compressions were done by laypersons and/or before arrival of the dispatched organized medical team.

Outcomes: Outcomes were graded on a nine-point scale according to the Grading of Recommendations, Assessment, Development and Evaluation (GRADE) approach^9^ through consensus discussion by the ILCOR First Aid Task Force as “critical” (4–6) or “important” (7–9).

Primary outcomes: Favorable neurological/functional outcome (critical)- as defined in studies at discharge, 30 days, 60 days, 180 days, and/or 1 year.

Secondary outcomes: Survival (critical) – defined as survival at discharge, 30 days, 60 days, 180 days, and/or 1 year. Unintentional physical injury (critical)- defined as any reported injury; the task force identified a priori rib fracture, sternum fracture and lung contusion as well as, any form of bleeding (dichotomous outcome; yes/no). Risk of injury (important) –defined as any reported complication not needing any active treatment/medications; the task force identified a priori aspiration (dichotomous outcome; yes/no).

It was anticipated that the reporting of outcomes varies between studies and that a meta-analysis might only be dichotomous (yes/no). A narrative summary of rates for each of these secondary outcomes is provided.

Study Designs: Included: Randomized controlled trials (RCTs) and non-randomized studies (non-randomized controlled trials, interrupted time series, controlled before-and-after studies, cohort studies). Excluded: Case series with less than 5 cases, unpublished studies, conference abstracts, trial protocols and animal studies.

Timeframe: All years and all languages were included if there was an English abstract.

### Search strategy and study selection

Search strings were developed by the Karolinska Institutet Library for the following databases: Medline (Ovid), Web of Science Core Collection (clarivate) and Cinahl (Ebsco). Databases were searched from their inception date until October 6, 2023. We reran the search on September 17, 2024, and no new articles were identified that fulfilled the criteria for inclusion. All search strategies can be found in Supplementary material.

Four authors (TD, FW, MO, JHP) independently screened titles and abstracts, and full texts. Screening was done using the software Rayyan.ai^10^ and a kappa value was calculated for inclusion following full text screening. Each article was screened by two authors and disparities were discussed until consensus. The main reasons for disparities was inclusion of a background article rather than an article containing the exposure and outcome of relevance. Reasons for full text exclusion, based on the selection criteria, were documented. Reference lists of all articles eligible for full text screening were assessed.

### Data collection

Two author independently extracted the following data: study design, study population and its characteristics as well as outcome measures (TD, FW).

### Risk of bias and certainty of evidence assessment

The GRADE approach was used to determine the certainty of evidence. The online available GRADEpro Guideline Development Tool (GDT) (https://www.gradepro.org/), was used to construct the GRADE evidence profile tables.^9^ The GRADE approach^11^ assesses the risk of bias, indirectness, imprecision, inconsistency and publication bias. The Risk of Bias In Non-randomized Studies of Interventions (ROBINS-I) tool^12^ and the online visualization tool (https://mcguinlu.shinyapps.io/robvis/) were used to independently assess bias by two reviewers (WTC, AMM). The final level of evidence was, in consensus, graded as high, moderate, low, or very low.

### Plan for data synthesis

We had planned, a priori, to perform random-effect meta-analysis using the generic inverse variance method for continuous data or the Mantel‐Haenszel method for dichotomous outcomes and assess heterogeneity by a visual inspection of the forest plot as well I^2^ statistic (heterogeneity considered significant if I^2^ > 60 %). However, due to limited numbers of identified articles, there were insufficient data to perform such.

A narrative summary of rates for each of the outcomes is provided.

## Results

### Study selection

**Fig. 1:**
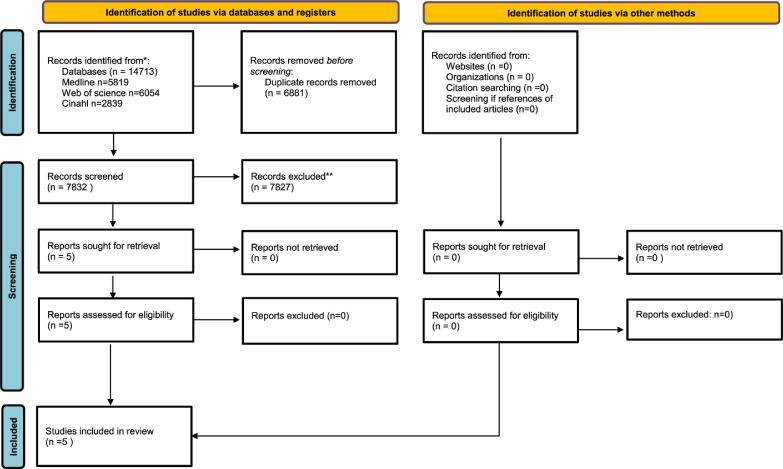
From: Page MJ, McKenzie JE, Bossuyt PM, Boutron I, Hoffmann TC, Mulrow CD, et al. The PRISMA 2020 statement: an updated guideline for reporting systematic reviews. BMJ 2021;372:n71. https://doi.org/10.1136/bmj.n71.

We identified 7832 references of which five met inclusion criteria (Fig. 1). A kappa value of 1.0 was calculated for inclusion following full text screening. No publications were added based on the review of reference lists of the full-text screened studies as well as review by citations of the article in PubMed, resulting in a total of five studies being included.^13^, ^14^, ^15^, ^16^, ^17^ Fig. 1 illustrates the PRISMA study selection diagram.

### Study characteristics

We identified five observational studies from two geographical areas in total including 1031 patients (Table 1). One study^13^ included 18 (23 %) patients under 18 years old. Two studies^15^, ^16^ assessed the implementation of a dispatcher-assisted CPR-protocol while the remaining three studies assessed injuries from CPR. All studies used medical records as their data source, two further used audio records^15^, ^17^ and three of the studies^13^, ^14^, ^17^ reviewed the medical records to identify injuries, and 1 included follow-up telephone interviews.^17^

**Table 1:** Study characteristics, definitions of terms and significant findings.

**Author, Year, Country**	**Study type**	**Inclusion criteria**	**Exclusion criteria**	**Number of patients**	**Definition of layperson**	**Definition of not in cardiac arrest**	**Definition of chest compressions**	**Significant findings on outcomes**
**White, 2010, Washington USA**	Observational cohort	1) Patient determined by emergency dispatcher to be unconscious and not breathing normally AND 2) Layperson CPR was not ongoing	Age less than 18 years old, cardiac arrest due to traumatic or mechanical respiratory mechanism, do-not-attempt- resuscitation orders.	313	Not specified.	Pulse (without public-access defibrillation) on EMS arrival.	Not specified.	Out of 1700 cases of DA-CPR, 762 (45 %) were not in cardiac arrest at EMS arrival. Out of 313, 83 (27 %) were discharged from ED, 2 (<1%) died in the ED and further 17 (5 %) died in-hospital. 22 (7 %) had pain or possible injury, 2 (<1%) had a rib fracture, none had internal bleeding, 1 (<1%) had other injury.
**Haley, 2011, Milwaukee USA**	Observational cohort	1) Chest compressions started by layperson AND 2) EMS record documented the presence of a palpable pulse, if the record documented a working assessment other than cardiac arrest, if EMS personnel stopped the layperson’s CPR and EMS personnel did not perform CPR, and/or if the EMS personnel otherwise noted somewhere on the EMS record that the victim was not in cardiac arrest	No chest compression by layperson, or only ventilations by the layperson.	77	Not specified.	Chest compression started by layperson in registry AND EMS record documented the presence of a palpable pulse, if the record documented a working assessment other than cardiac arrest, if EMS personnel stopped the layperson’s CPR and EMS personnel did not perform CPR, and/or if the EMS personnel otherwise noted somewhere on the EMS record that the victim was not in cardiac arrest.	Chest compressions by a person on scene at EMS arrival	Of all 672 victims with layperson CPR, 77 (12 %) was not in cardiac arrest. Out of 77 patients, 24 % was discharged to their home from the ED and 53 % was admitted to the ICU. 1 (1 %) patient had rhabdomyolysis.
**Moriwaki, 2012, Yokohama Japan**	Population- based observational case series	1) Patients who underwent CPR with chest compression by laypersons not belonging to any organized EMS, regardless of their training in CPR or whether advised by telephone CPR AND 2) Patients confirmed as non-OHCA and had to have not undergone any CPR by any organized EMS	1) Adult patients with OHCA diagnosed after contact with an EMS team. 2) Patients who received only a precordial thump, tapping of the back or abdominal thrust manoeuvre without chest compression.	26	Someone not part of an organized medical team regardless of their training and dispatcher-CPR.	CPR with chest compression by laypersons not belonging to any organized EMS, regardless of their training in CPR or whether advised by T-CPR AND the subjects also had to be confirmed as non-OHCA patients and had to have not undergone any CPR by any organized EMS such as an ambulance team.	Not only precordial thump, tapping on back or Heimlich.	Of all 910 victims with layperson CPR, 26 (3 %) were not in cardiac arrest. 9 (35 %) patients died of a cause (7 intracranial haemorrhage, 1 heart failure, 1 pneumonia) 3 (12 %) patients had a complication (1 tracheal bleeding, minor gastric laceration, chest pain). 3 (12 %) patients had symptom (undefined) that might be due to layperson CPR, such as chest pain at inspiration.
**Tanaka, 2014, Ishikawa Japan**	Observational cohort after implementation of a DA-CPR-protocol	Patients not in cardiac arrest on EMS arrival but DA-CPR attempted	Not specified.	417	Not specified.	Fire department protocols with ‘Yes’ on variable DA-CPR on patients not in cardiac arrest at EMS arrival.	Not specified.	Of 2747 victims with layperson CPR, 417 (15 %) were not in cardiac arrest. There was no reported complication for both groups of patients with and without cardiac arrest.
**Ng, 2021, Singapore**	Observational cohort after implementation of a DA-CPR prtocol	1) Dispatcher recognised it as cardiac arrest but the patient was later determined not to have been in cardiac arrest based on any one of A-D; A) absence of cardiac arrest on arrival of paramedics and in the ED B) absence of any treatment for cardiac arrest other than CPR C) absence of ventricular tachycardia or ventricular fibrillation D) no suggestive circumstances of drowning, electrical injury or overdose that could have resulted in a cardiac arrest but might have had spontaneous circulation following CPR.	Layperson CPR initiated without dispatchers' assistance	198	Not specified.	A case of false-positive recognition of cardiac arrest was defined as: where dispatchers recognised it as a cardiac arrest, but the patient was later determined not to have been in cardiac arrest. To ascertain that the patient did not actually have a cardiac arrest, he must meet all of the following criteria: absence of cardiac arrest on arrival of paramedics and in the ED, absence of any treatment for cardiac arrest other than CPR (i.e. defibrillation), absence of ventricular tachycardia (VT) or ventricular fibrillation (VF)) and no suggestive circumstances of an event, such as drowning, electrical injury and overdose that could have resulted in a cardiac arrest but might have had spontaneous circulation (ROSC) following CPR.	Layperson-initiated CPR upon the instructions of a dispatcher.	Out of 821 OHCA, 328 (40 %) did not have a cardiac arrest and out of them, 173 had DA-CPR and further 25 had layperson-initiated CPR. 100 were transported to the hospital, 14 (8 %) were discharged from ED, 6 (4 %) died in ED. 44 (25 %) died in 30 days, 25 (15 %) had readmission in 30 days. There was no reported complication due to CPR.

Definitions of layperson, chest compressions and whether the person was in cardiac arrest or not differed slightly between studies (Table 1). Characteristics for equity according to Cochranes checklist Progress Plus^18^, ^19^ was commonly lacking in studies, with only two studies^13^, ^15^ reporting ethnicity and location (health care facilities in 18 % and < 1 %), with male sex predominant in all studies (Supplementary material). The layperson relationship was only described in two studies and was reported as the arrest occurring at a nursing home, which might indirect imply that there is a relation between the layperson and victim, in one study in 9 %^13^ and that the layperson was a family member in 71 % in another study.^16^

Among all patients who received CPR from a layperson, approximately one in five was later assessed by EMS as not being in cardiac arrest (range: 3–40 %) (Table 1). Some studies included patients who had a detectable pulse upon EMS arrival, while others used broader criteria, such as the absence of ventricular fibrillation. Additionally, the duration of CPR varied between studies, with the majority of patients receiving compressions for less than five minutes (Supplementary material).

### Risk of bias

**Fig. 2:**
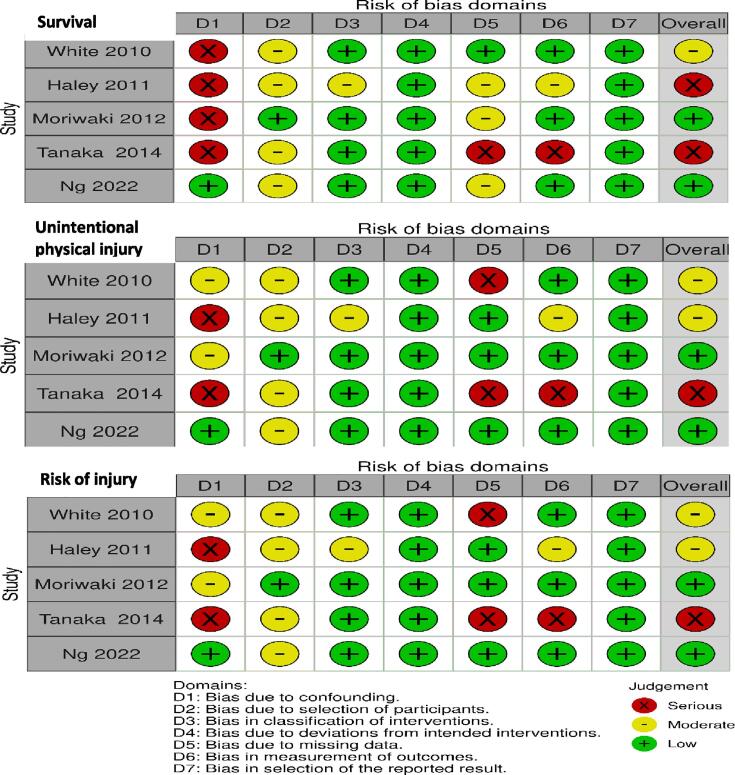
The risk of bias in non-randomized studies of interventions (ROBINS-I) tool^22^ for the outcomes visualized with the online tool (https://mcguinlu.shinyapps.io/robvis/).

Confidence in the estimate of effect, for the outcomes evaluated, was low due to risk of bias, imprecision due to small sample sizes and, in some cases, due to inconsistency (Fig. 2). Major types of biases identified were confounding, measurement of exposure, selection and missing data.

Selection bias related to the fact that it is fully possible that the person was in a cardiac arrest at the time of start of chest compressions. Measurement bias exits due to the varying methods of data collection across studies (e.g., medical records, audio recordings, and interviews). Missing data, particularly regarding patients not transported to hospitals, also introduced uncertainty since injuries such as a rib fracture or symptoms of chest pain might present later.

### Results of individual studies

One study^15^ compared patients not in cardiac arrest who had chest compressions with patients not in cardiac arrest who did not have chest compressions. This study reported no (n = 0) complications in either the 173 patients with layperson-CPR or the 130 patients who did not have CPR. Additionally, they did not find a difference in patients admitted to hospital from the ED as well as deaths in-hospital for those with chest compressions compared to those without (Table 2). The remaining four studies^13^, ^14^, ^16^, ^17^ reported outcomes in patients not in cardiac arrest given layperson-CPR without any control group.

**Table 2:** Outcome data from included studies.

Study	Number of patients	Mortality	Unintentional injury	Risk of unintentional injury	Others	Symptoms
White, 2010, Washington USA	247 out of 313 had outcome data.	20 (6 %) died – 3 in ED and 17 in hospital.	2 (0.6 %) rib fractures 1 (0.3 %) clavicle fracture 0 internal bleeding	1 (0.3 %) other injury (not further specified)	83 (27 %) were discharged from ED	22 (7 %) reported chest pain or discomfort
Haley, 2011, Milwaukee USA	72 out of 77 had outcome data.	−	−	1 (1 %) had rhabdomyolysis	18 (23 %) were discharged from ED	−
Moriwaki, 2012, Yokohama Japan	26	9 (35 %) died in hospital – 7 intracranial haemorrhage, 1 acute heart failure, 1 pneumonia.	1 (4 %) hematemesis – gastroscopy revealed minor gastric mucosal laceration 1 (4 %) tracheal bleeding found at intubation	−	5 (19 %) had mild neurological disorder, 2 (8 %) had severe neurological disorder and 4 (15 %) were in a vegetative state at discharge from hospital, all due to causative disorder not CPR	1 (4 %) had chest pain at inspiration
Tanaka, 2014, Ishikawa Japan	417	−	None reported	−	−	−
Ng, 2022, Singapore.	198	32 (18 %) for those with chest compressions compared to 13 (10 %) for those without (p-value 0.12 and 0.24). 6 (4 %) versus 1 (<1%) died in ED. 26 (15 %) versus 12 (9 %) died in hospital.	1 (<1%) had a rib fracture due to a fall 1 (<1%) had a chest hematoma		14 (8 %) were discharged from ED	1 (<1%) had chest pain but a normal chest xray
TOTAL*	**1031**	**61 (6 %) died in hospital but none was due to CPR according to authors of each study**	**7 (<1%)**	**2 (<1%)**	**−**	**24 (2 %)**

*Note: Percentage calculated based on the total number of patients in the study regardless if they had missing data or not.

For the primary outcome survival with favourable neurological outcome, no data was found but for the secondary outcome survival, no deaths due to CPR were reported, but 61 (6 %) patients were admitted to the hospital but died before discharge from hospital. No studies reported neurological outcome in survivors or the outcome aspiration.

Across the five studies on 1031 patients not in a cardiac arrest, less than 1 % (n = 7) of patients experienced unintentional physical injury, less than 1 % (n = 2) had risk of unintentional injuries and a further 24 (2 %) had symptoms such as chest pain or discomfort (Table 2).

#### Unintentional physical injury

For the outcome of “unintentional physical injury” we identified very low certainty evidence (downgraded for risk of bias and imprecision) from four observational studies^14^, ^15^, ^16^, ^17^ enrolling 954 patients who were not in cardiac arrest and received CPR by lay rescuers outside the hospital.

Across all studies we found that < 1 % (n = 4) of the patients had a reported fracture (ribs and clavicle) and an incidence of internal bleeding of < 1 % (one case)^14^, asymptomatic tracheal bleeding < 1 % (one case) and a chest hematoma < 1 % (one case).^15^

#### Risk of injury

For the important outcome of “risk of injury” we identified very low certainty evidence (downgraded for risk of bias and imprecision) from two observational studies^13^, ^17^ enrolling 691 patients who were not in cardiac arrest. Data from both studies revealed an incidence of rhabdomyolysis of < 1 % (one case)^13^ and non-specified other injury < 1 % (one case).^17^

#### Other outcomes

We identified very low certainty evidence (downgraded for imprecision) from three observational studies^14^, ^15^, ^17^ enrolling 537 patients who were not in cardiac arrest regarding symptoms. Data from the three studies taken together revealed that 24 (4 %) of the patients reported having chest pain or discomfort.

### Certainty of the evidence

For all outcomes, the certainty of the evidence was very low since it was downgraded by one level for indirectness, as well as one level for imprecision (Supplementary material). Further downgrading of certainty was due to the fact that there were too few studies included to generate funnel plots or judge publication bias.

## Discussion

Early CPR is an important factor in the chain of survival for OHCA. As a result, resuscitation councils internationally have focussed their efforts on promoting layperson CPR until trained providers arrive.^20^ A recognised barrier to layperson CPR is the concern that it may cause an unintentional injury, resulting in a reluctance to commence resuscitation.

Within this systematic review we were not able to perform meta-analysis since only one study actually included patients not in cardiac arrest and not receiving chest compressions. Instead we performed a narrative reporting of unintentional physical injuries from chest compressions in patients not in cardiac arrest. The incidence of layperson-CPR in non-cardiac arrest patients and quantified the risk of injuries when laypersons perform CPR on patients who are not in cardiac arrest. This risk is low, with less than 1 % of patients being injured or having a risk of injury following chest compressions provision in this setting. Since our outcomes were rare, we downgraded certainty due to imprecision, however, the current review further supports the recommendations of the 2020 ILCOR statement: that ‘laypersons can initiate CPR for presumed cardiac arrest without concerns of harm to patients not in cardiac arrest’.

The incidence of CPR-related injuries in the papers included in this review was much lower than in studies where patients who had been in cardiac arrest and received CPR were assessed for injuries.^21^ Of note is that the lack of follow-up in the included studies might introduce bias. For example, rib fractures occur in nearly 50 % of patient in cardiac arrest.^2^ This is likely due to a longer duration of CPR being delivered to patients who are actually in cardiac arrest, the frequent use of mechanical compressions and potential bias as the studies on patients who had been in cardiac arrest included autopsy studies.

Few aspects of equity were reported in studies; the use of a structure such as Cochranes PROGRESS Plus might increase reporting. There was a roughly equal portion of men and women in the studies. However, it was noted, in three studies, that the layperson often had some kind of relation to the victim, either as a family member or personnel at a nursing home. They might both fear causing an injury and prioritize survival.

Limitations with the review include that none of the five studies were prospective or randomised and the studies were too heterogeneous to perform a meta-analysis. Further, there is no consensus on which injuries that is caused by chest compressions and some of the findings such as rhabdomyolysis has only been described in a case report previously.^22^ In addition, it is possible that some of the included patients may have had a short duration of cardiac arrest, and then achieved ROSC, this may be particularly prevalent amongst patients with opiate overdose induced collapse, however none of the included studies included patients with prolonged resuscitation. Further, only one study included children. It is likely that the incidence of rib fractures and other CPR related injuries rises as patient age and frailty increase, but none of the papers specifically addressed this patient group. Three of the included studies employed different dispatcher protocols for CPR and it might be possible to use the result to support emergency medical dispatchers or telecommunicators in their efforts to provide telephone assisted CPR instructions in suspected cardiac arrest calls, but the first aid task force felt that it is beyond the scope of this review.

Finally, attitudes towards performing CPR as a layperson might differ between cultures, the included studies were from US and Asia, but none from other continents.

The low risk of injury to patients not in cardiac arrest who receive short durations of CPR should be balanced against the greater number of collapsed patients who are in cardiac arrest and whose outcomes are improved by receiving layperson-CPR.

## Conclusions

In patients initially receiving CPR by a layperson and who later determine by medical professionals to not be in cardiac arrest the rates of injuries from chest compressions are low. International guidelines may recommend that if there is any suspicion about whether a patient is in cardiac arrest then chest compressions should be commenced.

## Collaborators

Members of the International Liaison Committee on Resuscitation First Aid Task Force who met criteria as a collaborator include: David Berry, Richard Bradley, Pascal Cassan, Nathan Charlton, Diana Cimpoesu, Gustavo Flores-Bauer, Craig Goolsby, Barry Klaassen, Amy Kule, Jorien Laermans, Finley MacNeil, Daniel Meyran, Aaron Orkin, Jessica Rogers, Eunici Singletary, Willem Stassen, Lim Swee Han, Kaushila Thikashila.

## CRediT authorship contribution statement

**Frances Williamson:** Writing – review & editing, Writing – original draft, Methodology, Formal analysis, Conceptualization. **Pek Jen Heng:** . **Masashi Okubo:** . **Abel Martinez Mejias:** . **Wei-Tien Chang:** Writing – review & editing, Methodology, Conceptualization. **Matthew Douma:** Writing – review & editing, Methodology, Conceptualization. **Jestin Carlson:** Writing – review & editing, Methodology, Conceptualization. **James Raitt:** Writing – review & editing, Methodology, Conceptualization. **Therese Djärv:** Writing – review & editing, Writing – original draft, Validation, Supervision, Project administration, Methodology, Formal analysis, Data curation, Conceptualization.

## Funding

None.

## Declaration of competing interest

The authors declare that they have no known competing financial interests or personal relationships that could have appeared to influence the work reported in this paper.
