# Supplementary material for: Does delivering chest compressions to patients who are not in cardiac arrest cause unintentional injury? A systematic review
Source: Resusc Plus. 2024 Nov 23;20:100828. doi: 10.1016/j.resplu.2024.100828 (PMC11625224; doi:10.1016/j.resplu.2024.100828)
Supplement: Supplementary Data 1 [file mmc1.docx]

Supplementary Material: Search Strategy (Original search on 6 October 2023, repeat search on 17 September 2024)

1. Medline

| Interface: Ovid MEDLINE(R) ALL  Date of Search: 2024-09-17  Number of hits: 5819  Comment: In Ovid, two or more words are automatically searched as phrases; i.e. no quotation marks are needed | Field labels   - exp/ = exploded MeSH term - / = non exploded MeSH term - .ti,ab,kf. = title, abstract and author keywords - adjx = within x words, regardless of order - * = truncation of word for alternate endings |
| --- | --- |
| Ovid MEDLINE(R) ALL <1946 to September 16, 2024>   \| 1 \| exp Cardiopulmonary Resuscitation/ \| 23536 \| \| --- \| --- \| --- \| \| 2 \| (CPR or CPR-* or DA-CPR or cardiopulmonary resuscitation* or Cardio-Pulmonary Resuscitation* or cardio pulmonary resuscitation* or mouth to mouth resuscitation* or Mouth-to-Mouth Resuscitation* or cardiac life support* or code blue*).ti,ab,kf. \| 30291 \| \| 3 \| Out-of-Hospital Cardiac Arrest/ \| 7862 \| \| 4 \| (OH-CPA or OHCA or OOHCA or out of hospital cardiac* or out of hospital cardiopulmonary* or out of hospital cardio pulmonary* or out of hospital cardio-pulmonary* or out-of-hospital cardiac* or out-of-hospital cardiopulmonary* or out-of-hospital cardio pulmonary* or out-of-hospital cardio-pulmonary* or out of hospital heart* or out-of-hospital heart*).ti,ab,kf. \| 10097 \| \| 5 \| Heart Massage/ or Chest Wall Oscillation/ \| 3575 \| \| 6 \| (Cardiac Massage* or Heart Massage* or Chest Wall Oscillation* or Chest Compression* or Chest Wall Compression*).ti,ab,kf. \| 6886 \| \| 7 \| exp Heart Arrest/ \| 58580 \| \| 8 \| (Heart Arrest* or Cardiac Arrest* or Asystole* or Cardiopulmonary Arrest* or Cardio-pulmonary Arrest* or Cardio pulmonary Arrest*).ti,ab,kf. \| 54945 \| \| 9 \| or/1-8 \| 103826 \| \| 10 \| Volunteers/ or Hospital Volunteers/ \| 12387 \| \| 11 \| (Volunteer* or Voluntary Worker* or Voluntary Personnel* or Volunteer Worker* or Volunteer Personnel* or Volunteerism* or Untrained or Volunteer responder* or Voluntary responder*).ti,ab,kf. \| 238122 \| \| 12 \| (bystander* or first responder* or layperson* or layperson* or lay rescuer* or layrescuer* or laypeople or lay people or nonprofessional* or non professional* or witness*).ti,ab,kf. \| 62351 \| \| 13 \| or/10-12 \| 304604 \| \| 14 \| 13 and 9 \| 5819 \| | |

2. Web of Science Core Collection

| Interface: Clarivate Analytics  Editions = A&HCI , ESCI , SCI-EXPANDED , SSCI  Date of Search: 2024-09-17  Number of hits: 6054 | Field labels   - TS/Topic = title, abstract, author keywords and Keywords Plus - NEAR/x = within x words, regardless of order - * = truncation of word for alternate endings   Note: the *Exact search*-function was used for all the searches |
| --- | --- |
| \| # \| Search Query \| Results \| \| --- \| --- \| --- \| \| 1 \| TS=(CPR OR CPR-* OR DA-CPR OR "cardiopulmonary resuscitation*" OR "Cardio-Pulmonary Resuscitation*" OR "cardio pulmonary resuscitation*" OR "mouth to mouth resuscitation*" OR "Mouth-to-Mouth Resuscitation*" OR "cardiac life support*" OR "code blue*" ) \| 40019 \| \| 2 \| TS=(OH-CPA OR OHCA OR OOHCA OR "out of hospital cardiac*" OR "out of hospital cardiopulmonary*" OR "out of hospital cardio pulmonary*" OR "out of hospital cardio-pulmonary*" OR "out-of-hospital cardiac*" OR "out-of-hospital cardiopulmonary*" OR "out-of-hospital cardio pulmonary*" OR "out-of-hospital cardio-pulmonary*" OR "out of hospital heart*" OR "out-of-hospital heart*" ) \| 11593 \| \| 3 \| TS=("Cardiac Massage*" OR "Heart Massage*" OR "Chest Wall Oscillation*" OR "Chest Compression*" OR "Chest Wall Compression*" ) \| 7369 \| \| 4 \| TS=("Heart Arrest*" OR "Cardiac Arrest*" OR Asystole* OR "Cardiopulmonary Arrest*" OR "Cardio-pulmonary Arrest*" OR "Cardio pulmonary Arrest*" ) \| 64052 \| \| 5 \| #1 OR #2 OR #3 OR #4 \| 85762 \| \| 6 \| TS=(Volunteer* OR "Voluntary Worker*" OR "Voluntary Personnel*" OR "Volunteer Worker*" OR "Volunteer Personnel*" OR Volunteerism* OR Untrained OR "Volunteer responder*" OR "Voluntary responder*" ) \| 266655 \| \| 7 \| TS=(bystander* OR "first responder*" OR layperson* OR "layperson*" OR "lay rescuer*" OR layrescuer* OR laypeople* OR "lay people*" OR nonprofessional* OR "non professional*" OR witness* ) \| 120467 \| \| 8 \| #6 OR #7 \| 385877 \| \| 9 \| #5 AND #8 \| 6054 \| | |

3. Cinahl

| Interface: Ebsco  Date of Search: 2024-09-17  Number of hits: 2839 | Field labels   - MH+ = exploded Cinahl Heading - MH = non exploded Cinahl Heading - TI = title - AB = abstract - Nx = within x words, regardless of order - * = truncation of word for alternate endings |
| --- | --- |
| \| # \| Query \| Results \| \| --- \| --- \| --- \| \| S13 \| S8 AND S12 \| 2,839 \| \| S12 \| S9 OR S10 OR S11 \| 80,546 \| \| S11 \| ((TI bystander* OR AB bystander*) OR (TI "first responder*" OR AB "first responder*") OR (TI layperson* OR AB layperson*) OR (TI "layperson*" OR AB "layperson*") OR (TI "lay rescuer*" OR AB "lay rescuer*") OR (TI layrescuer* OR AB layrescuer*) OR (TI laypeople OR AB laypeople) OR (TI "lay people" OR AB "lay people") OR (TI nonprofessional* OR AB nonprofessional*) OR (TI "non professional*" OR AB "non professional*") OR (TI witness* OR AB witness*)) \| 18,698 \| \| S10 \| ((TI Volunteer* OR AB Volunteer*) OR (TI "Voluntary Worker*" OR AB "Voluntary Worker*") OR (TI "Voluntary Personnel*" OR AB "Voluntary Personnel*") OR (TI "Volunteer Worker*" OR AB "Volunteer Worker*") OR (TI "Volunteer Personnel*" OR AB "Volunteer Personnel*") OR (TI Volunteerism* OR AB Volunteerism*) OR (TI Untrained OR AB Untrained) OR (TI "Volunteer responder*" OR AB "Volunteer responder*") OR (TI "Voluntary responder*" OR AB "Voluntary responder*")) \| 53,197 \| \| S9 \| (MH “Volunteer workers”) OR (MH “Volunteer experiences”) \| 16,303 \| \| S8 \| S1 OR S2 OR S3 OR S4 OR S5 OR S6 OR S7 \| 46,523 \| \| S7 \| ((TI "Heart Arrest*" OR AB "Heart Arrest*") OR (TI "Cardiac Arrest*" OR AB "Cardiac Arrest*") OR (TI Asystole* OR AB Asystole*) OR (TI "Cardiopulmonary Arrest*" OR AB "Cardiopulmonary Arrest*") OR (TI "Cardio-pulmonary Arrest*" OR AB "Cardio-pulmonary Arrest*") OR (TI "Cardio pulmonary Arrest*" OR AB "Cardio pulmonary Arrest*")) \| 21,479 \| \| S6 \| (MH "Heart Arrest+") \| 22,973 \| \| S5 \| ((TI "Cardiac Massage*" OR AB "Cardiac Massage*") OR (TI "Heart Massage*" OR AB "Heart Massage*") OR (TI "Chest Wall Oscillation*" OR AB "Chest Wall Oscillation*") OR (TI "Chest Compression*" OR AB "Chest Compression*") OR (TI "Chest Wall Compression*" OR AB "Chest Wall Compression*")) \| 2,743 \| \| S4 \| (MH "Heart Massage") \| 627 \| \| S3 \| ((TI OH-CPA OR AB OH-CPA) OR (TI OHCA OR AB OHCA) OR (TI OOHCA OR AB OOHCA) OR (TI "out of hospital cardiac*" OR AB "out of hospital cardiac*") OR (TI "out of hospital cardiopulmonary*" OR AB "out of hospital cardiopulmonary*") OR (TI "out of hospital cardio pulmonary*" OR AB "out of hospital cardio pulmonary*") OR (TI "out of hospital cardio-pulmonary*" OR AB "out of hospital cardio-pulmonary*") OR (TI "out-of-hospital cardiac*" OR AB "out-of-hospital cardiac*") OR (TI "out-of-hospital cardiopulmonary*" OR AB "out-of-hospital cardiopulmonary*") OR (TI "out-of-hospital cardio pulmonary*" OR AB "out-of-hospital cardio pulmonary*") OR (TI "out-of-hospital cardio-pulmonary*" OR AB "out-of-hospital cardio-pulmonary*") OR (TI "out of hospital heart*" OR AB "out of hospital heart*") OR (TI "out-of-hospital heart*" OR AB "out-of-hospital heart*")) \| 5,872 \| \| S2 \| ((TI CPR OR AB CPR) OR (TI CPR-* OR AB CPR-*) OR (TI DA-CPR OR AB DA-CPR) OR (TI "cardiopulmonary resuscitation*" OR AB "cardiopulmonary resuscitation*") OR (TI "Cardio-Pulmonary Resuscitation*" OR AB "Cardio-Pulmonary Resuscitation*") OR (TI "cardio pulmonary resuscitation*" OR AB "cardio pulmonary resuscitation*") OR (TI "mouth to mouth resuscitation*" OR AB "mouth to mouth resuscitation*") OR (TI "Mouth-to-Mouth Resuscitation*" OR AB "Mouth-to-Mouth Resuscitation*") OR (TI "cardiac life support*" OR AB "cardiac life support*") OR (TI "code blue*" OR AB "code blue*”)) \| 13,435 \| \| S1 \| (MH "Resuscitation, Cardiopulmonary+") \| 17,484 \| | |

Supplementary Material: Characteristics for Equity According to Cochrane Checklist Progress Plus

| **Author, Year, Country** | **Race/ethnicity/ culture/language** | **Occupation** | **Gender** | **Religion** | **Education** | **Socio-economic status** | **Social capital** | **Discriminating personal characteristics** | **Relationships** | **Time-dependent relations** |
| --- | --- | --- | --- | --- | --- | --- | --- | --- | --- | --- |
| **White, 2010,**  **Washington USA** |  |  | Male 367 (54%) |  |  |  |  |  |  | CPR on average for 91 seconds |
| **Haley, 2011, Milwaukee USA** | White 43 (56%), African-American 20 (26%), Other/NA 14 (18%) | 18 (23%) patients under 18 years old | Male 39 (51%) |  |  |  |  | 14 (18%) at a healthcare facility and further 7 (9%) at a nursing home | 7 (9%) at a nursing home | EMS arrival within 5 min (68%) and beyond 10 min (2%) |
| **Moriwaka, 2012, Yokohama Japan** |  |  | Male 15 (58%) |  |  |  |  |  |  | CPR on average for 6.5 min |
| **Tanaka, 2014, Ishikawa Japan** |  |  |  |  |  |  |  | 1396 (75.1%) at home | 1294 (71%) of the bystander were family |  |
| **Ng, 2022, Singapore** | Chinese 126 (73%), Malay 21 (12%), Indian 11 (6%), Other 15 (9%) |  | Male 91 (53%) |  |  |  |  | 86 (49.7%) at home, 1 (0.6%) healthcare facility |  |  |

Supplementary Material: Certainty Assessment

| **Certainty assessment** | | | | | | | **Number of patients** | | **Effect** | | **Certainty** | **Importance** |
| --- | --- | --- | --- | --- | --- | --- | --- | --- | --- | --- | --- | --- |
| **Number of studies** | **Study design** | **Risk of bias** | **Inconsistency** | **Indirectness** | **Imprecision** | **Other considerations** | **Chest compressions** | **No chest compressions** | **Relative (95% CI)** | **Absolute (95% CI)** |  |  |
| All outcomes | | | | | | | | | | | | |
| 1 | non-randomised studies | very serious | serious | serious | serious | none | 0/273 (0.0%) | 0/130 (0.0%) | not estimable |  | ⨁◯◯◯ Very low | CRITICAL |
| Mortality | | | | | | | | | | | | |
| 3 | non-randomised studies | serious | serious | very serious | serious | none | 61/1031 (5.9%) | - | not estimable |  | ⨁◯◯◯ Very low | CRITICAL |
| **Unintentional injury** | | | | | | | | | | | | |
| 3 | non-randomised studies | very serious | serious | very serious | very serious | all plausible residual confounding would reduce the demonstrated effect dose response gradient | 7/954 (0.7%) | - | not estimable |  | ⨁◯◯◯ Very low | CRITICAL |
| **Risk of injury** | | | | | | | | | | | | |
| 2 | non-randomised studies | very serious | very serious | very serious | very serious | all plausible residual confounding would reduce the demonstrated effect dose response gradient | 2/691 (0.3%) | - | not estimable |  | ⨁◯◯◯ Very low | IMPORTANT |

**CI:** confidence interval
